# How do tobacco control policies work in low-income and middle-income countries? A realist synthesis

**DOI:** 10.1136/bmjgh-2022-008859

**Published:** 2022-11-08

**Authors:** Pragati Bhaskar Hebbar, Vivek Dsouza, Upendra Bhojani, Nuggehalli Srinivas Prashanth, Onno CP van Schayck, Giridhara R Babu, Gera E Nagelhout

**Affiliations:** 1 Cluster on Chronic conditions and public policies, Institute of Public Health Bengaluru, Bangalore, Karnataka, India; 2 Department of Health Promotion, Maastricht University, Maastricht, Netherlands; 3 Cluster on Health equity, Institute of Public Health Bengaluru, Bangalore, Karnataka, India; 4 Department of Family Medicine, Care and Public Health Research Institute (CAPHRI), Maastricht University Faculty of Health Medicine and Life Sciences, Maastricht, Netherlands; 5 Department of Population Medicine, College of Medicine, QU Health, Qatar University, Doha, Qatar

**Keywords:** Health systems evaluation, Health policy, Health education and promotion, Control strategies

## Abstract

**Background:**

The burden of tobacco use is disproportionately high in low- and middle-income countries (LMICs). There is scarce theorisation on what works with respect to implementation of tobacco control policies in these settings. Given the complex nature of tobacco control policy implementation, diversity in outcomes of widely implemented policies and the defining role of the context, we conducted a realist synthesis to examine tobacco control policy implementation in LMICs.

**Methods:**

We conducted a systematic realist literature review to test an initial programme theory developed by the research team. We searched EBSCOHost and Web of Science, containing 19 databases. We included studies on implementation of government tobacco control policies in LMICs.

**Results:**

We included 47 studies that described several contextual factors, mechanisms and outcomes related to implementing tobacco control policies to varying depth. Our initial programme theory identified three overarching strategies: awareness, enforcement, and review systems involved in implementation. The refined programme theory identifies the plausible mechanisms through which these strategies could work. We found 30 mechanisms that could lead to varying implementation outcomes including normalisation of smoking in public places, stigmatisation of the smoker, citizen participation in the programme, fear of public opposition, feeling of kinship among violators and the rest of the community, empowerment of authorised officials, friction among different agencies, group identity among staff, shared learning, manipulation, intimidation and feeling left out in the policy-making process.

**Conclusions:**

The synthesis provides an overview of the interplay of several contextual factors and mechanisms leading to varied implementation outcomes in LMICs. Decision-makers and other actors may benefit from examining the role of one or more of these mechanisms in their particular contexts to improve programme implementation. Further research into specific tobacco control policies and testing particular mechanisms will help deepen our understanding of tobacco control implementation in LMICs.

**PROSPERO registration number:**

CRD42020191541.

**WHAT IS ALREADY KNOWN ON THIS TOPIC**

- Tobacco control research over the past decades has evolved to identify effective policy measures, but how do these policies work in different settings needs to be further unpacked

**WHAT THIS STUDY ADDS**

- This realist synthesis deciphers underlying mechanisms that might be at play in the implementation of policies in complex real world resource limited settings that bear a large brunt of death and disease due to tobacco use.

**HOW THIS STUDY MIGHT AFFECT RESEARCH, PRACTICE OR POLICY**

- Theorisation and explanation of implementation of tobacco control policies will supplement existing practices and guide future policy implementation efforts by anticipating barriers and designing policies to avoid or address them.

## Background

Despite growing interest in unpacking the black box of policy implementation, there is scarce research on the social complexity in which implementation is embedded.^1^ Tobacco use and its control are complex social phenomena due to the diversity of actors, competing interests, and the politics and economics of sales versus the health costs.^2^ The burden of tobacco use and tobacco-related deaths is disproportionately high in low- and middle-income countries (LMICs). Around 77% of smoking-related deaths and 89% of secondhand smoke-related deaths occur in LMICs.^3^ Globally, the WHO’s Framework Convention on Tobacco Control (WHO FCTC) provides an overarching policy framework for countries to implement tobacco control programmes.^4^ The FCTC marked a milestone in consolidating various efforts at innovative programme design for tobacco control that has been active over the last two decades and has triggered several policy initiatives in member countries. This has aligned with the global efforts at methodological advancements and field-building within the implementation science community that has focused on advancing our understanding of the contextual nature of change.^5^ Indeed, despite global and national policies for tobacco control, implementation remains variable across and within countries, necessitating the need to improve the understanding of tobacco control policy implementation.^6^

Policy studies over the past several decades have attempted to explain the process of implementation as if it proceeds in successive stages.^7^ The implementation or execution of the policy comes at a much later stage in these stages and models with agenda setting, policy formulation, etc preceding it. Implementation is typically classified within top-down or bottom-up arrangements, with some integrating a more hybrid form/typology,^7^ but with an overall lack of a theoretical framework explaining the implementation of tobacco control policies.^3^ The stage based approach falls short in embracing the complexity of the implementation process that can sometimes take convoluted paths. For the purpose of this study, we refer to the process of implementation as: What happens between policy expectations and (perceived) policy results (paraphrasing Ferman, 1990).^7^ Another comprehensive definition of implementation is formulated by Mazmanian and Sabatier (1983): Implementation is the carrying out of a basic policy decision, usually incorporated in a statute but which can also take the form of important executive orders or court decisions. Ideally, that decision identifies the problem(s) to be addressed, stipulates the objective(s) to be pursued, and in a variety of ways, ‘structures’ the implementation process.^7^

Realist synthesis has gained wider adoption since 2002.^8^ It seeks to explain what works or does not work, for whom, and under what circumstances.^8–11^ Realist synthesis applies the realist approach philosophically rooted in realism to explain the successes, failures or varying eventualities in-between by studying the interactions between contexts (C), mechanisms (M) and outcomes (O). It uses the CMO heuristic to elicit and explain the main ideas related to the programme under study. This is termed as the programme theory. The CMO configurations explain how the programme was supposed to operate vs how it has operated in different situations.^11^

Given the complex nature of tobacco control, diversity in the outcomes of widely implemented policies, dearth of theorisation in the body of literature on implementation of tobacco control policies, and the defining role of the context, we conducted a realist synthesis to examine tobacco control policy implementation in LMICs. Our research question for this realist synthesis was: What are the facilitators and barriers to implementing tobacco control policies in LMICs?

## Methods

The reporting standards for realist synthesis developed by the RAMESES project was used (see online supplemental file 1).^11^

10.1136/bmjgh-2022-008859.supp1 Supplementary data

### Initial program theory development

**Figure 1:**
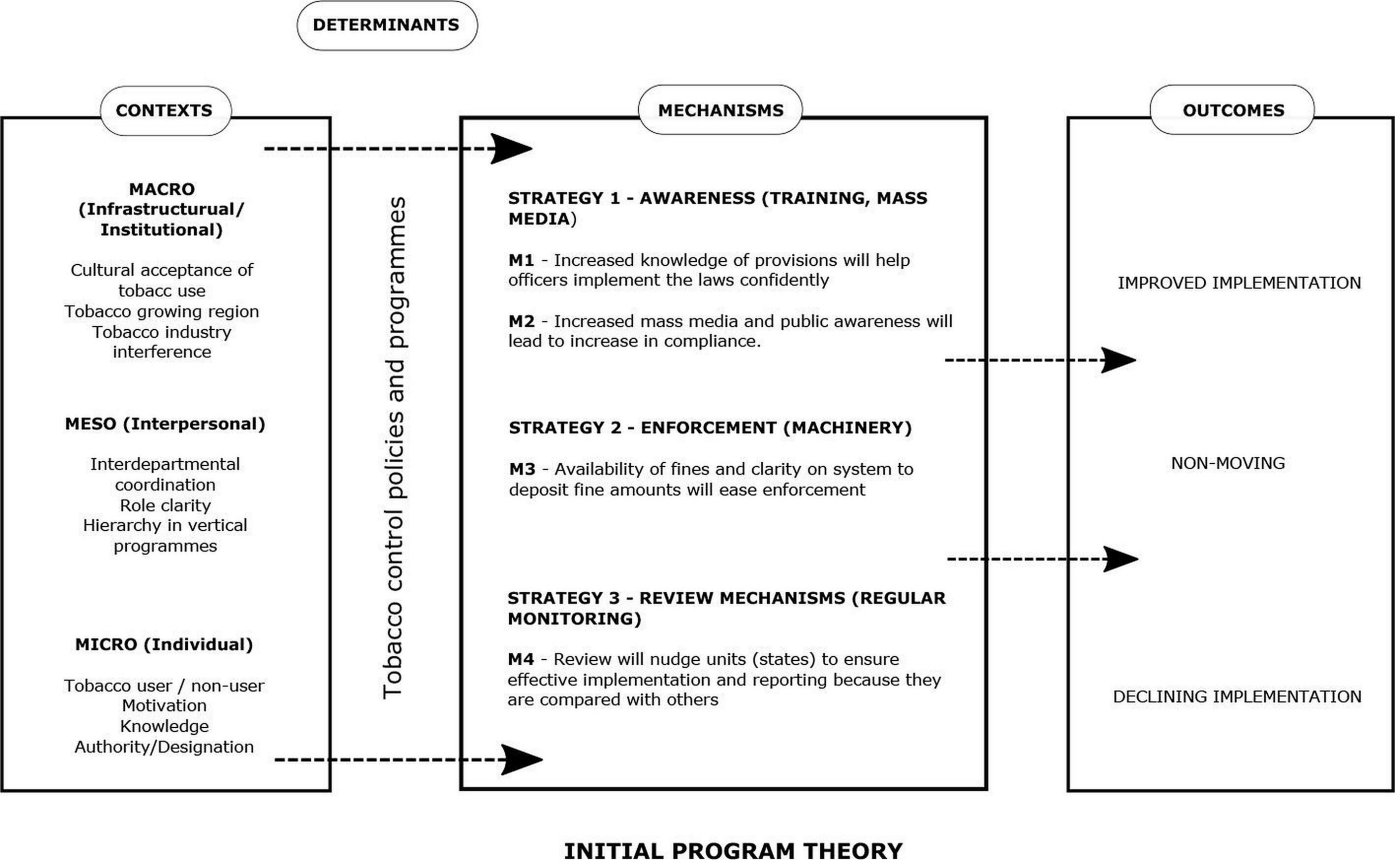
Schematic of the initial programme theory.

The WHO-FCTC and MPOWER provide a comprehensive structure and guidance for the diverse tobacco control policies. Realist sythesis is a theory-driven literature review methodology to explain what works for whom under what circumstances and begins with initial programme theories (IPT) that can be supported, refuted or refined based on the literature.^9^ To inspire the development of an IPT, we explored several theories related to the implementation of policies during the initial phase of this study. These theories and frameworks helped conceptualise and categorise contextual factors into microevel, mesoevel and macrolevel; articulate mechanisms under three strategies, and comprehend the interconnected contexts and mechanisms leading to different implementation outcomes.^12–17^ Most of the theories conceptualise implementation from different perspectives and different levels (institutional, organisational and individual) but lacked the specific factors such as the presence of tobacco industry interference, the underlying sociocultural climate, political commitment and institutional capacity in the implementation of tobacco control. The recent Hoe *et al*^17^ framework captures these specificities related to tobacco control and explains their interconnectedness affecting implementation. We developed a schematic eliciting an IPT of implementation to guide the literature review (see figure 1). Review of policy and programme documents, tobacco control research and advocacy experience of authors based in LMICs (PBH and UB) and inputs from policy advocates and researchers helped in conceptualising the IPT. The IPT has macrocontextual, mesocontextual and microcontextual factors and mechanisms related to three strategies of awareness, enforcement and assess/review, leading to varied (improved, stagnant or declining) implementation levels of tobacco control policies. These three strategies—to educate (raise awareness), enforce and assess—have been proposed by the Campaign for Tobacco Free Kids to explain the implementation of tobacco control polices at a legal development programme capacity building session in 2016.^16^ The strategy of awareness refers to awareness among the general public about harms of tobacco as well as tobacco control laws and the awareness of the laws among officials who are authorised to implement the tobacco control laws. The strategy of enforcement refers to application or execution of the law by the authorised officials. Lastly, the strategy of assessment or review systems refers to checkpoints within the system such as monthly meetings or quarterly meetings where the implementation of the law is reviewed. Through this realist synthesis, we aim to refine and expand the IPT to explain the process of implementation specifically in LMICs.

### Search strategy

We followed the five steps proposed by Pawson *et al*^8^ for conducting a realist synthesis, which is a type of systematic review designed for complex policy interventions, that is (1) clarifying scope, (2) searching for evidence, (3) appraisal of primary studies and extraction of data, (4) synthesising evidence and drawing conclusion, and (5) disseminate, implement and evaluate. We conducted initial searches in various databases with multiple combinations of the search terms to finalise a search strategy and databases yielding relevant results. A final literature search was conducted in April 2020 and updated in June 2022 through 13 scientific databases in EBSCOHost—APA PsycArticles, CINAHL, EconLit, ERIC, Library, Information Science & Technology Abstracts, APA PsycInfo, SocINDEX, MEDLINE, APA PsycBooks, eBook Collection, OpenDissertations, eBook Academic Collection, and Health and Psychosocial Instruments, and six scientific databases in Web of Science—Science Citation Index expanded (SCI-EXPANDED) Social Sciences Citation Index (SSCI) Arts and Humanities Citation Index (A&HCI) Conference Proceedings Citation Index—cience (CPCI-S) Conference Proceedings Citation Index-Social Science and Humanities (CPCI-SSH) Emerging Sources Citation Index (ESCI). The following search terms were used: [“Tobacco control” AND “Implementation” AND (“developing countries” OR “developing nations” OR “third world” OR “low income countries”)]. The detailed search string is provided in online supplemental file 2). The searches yielded 2651 citations (after the removal of 1162 duplicates).

10.1136/bmjgh-2022-008859.supp2 Supplementary data

### Selection of studies

**Figure 2:**
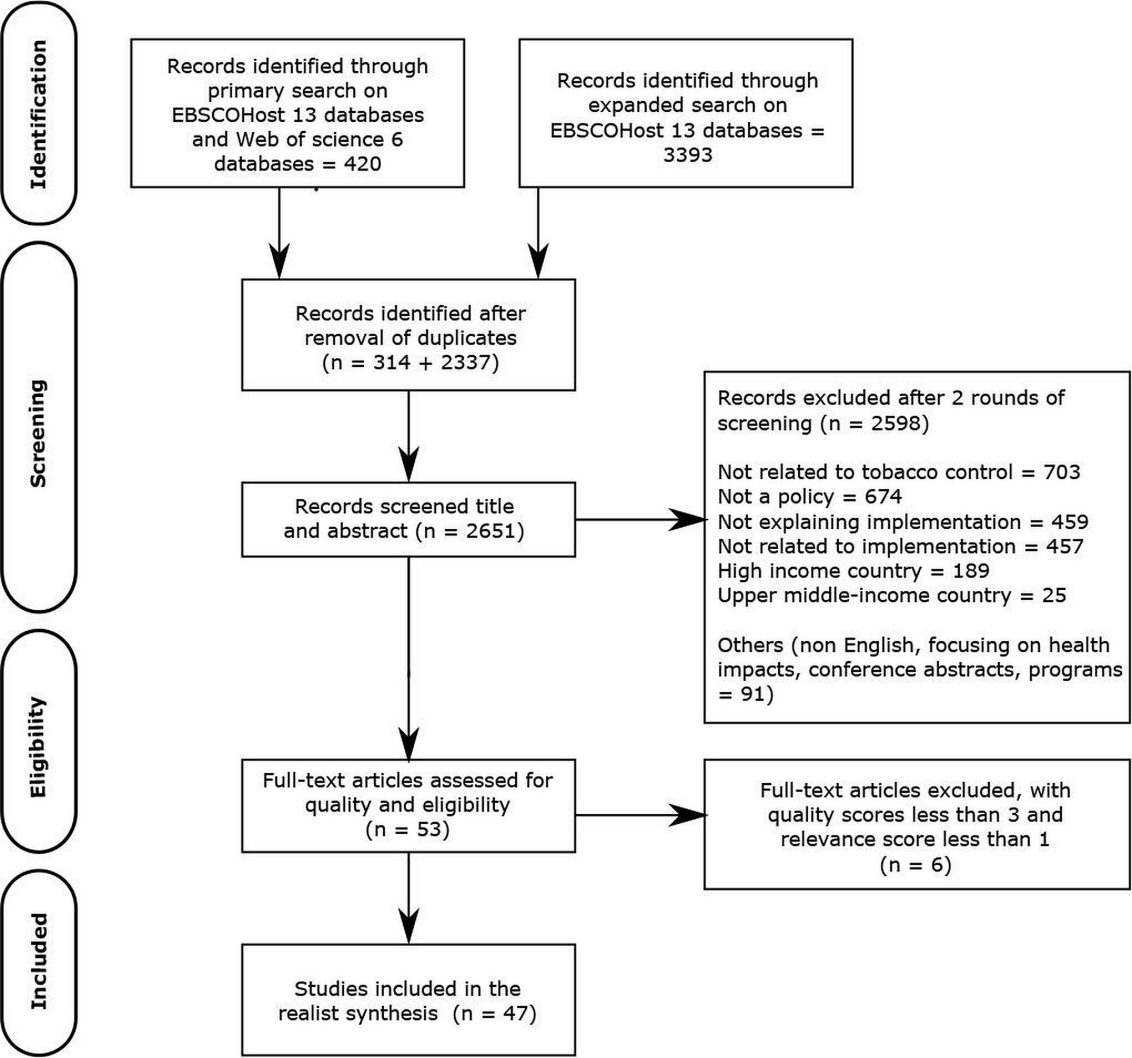
A detailed systematic review illustrated through the PRISMA flow diagram. PRISMA, Preferred Reporting Items for Systematic Reviews and Meta-Analyses.

We used the Preferred Reporting Items for Systematic Reviews and Meta-Analyses guideline to report the selection of studies (see figure 2). PBH developed the inclusion and exclusion criteria were which was reviewed and agreed on by the research team. Two reviewers PBH and VD independently screened titles and abstracts of all the citations with an intercoder difference of Cohen’s kappa=0.58 and arrived at a subset of articles. Differences between the two reviewers were resolved through multiple reflexive discussions where PBH and VD would explain their understanding and reasoning of coding and reach consensus. Owing to the large subset of articles the team revisited and added to the inclusion and exclusion criteria (point 4 of inclusion criteria and points 4–7 of exclusion criteria). PH conducted the second round of screening using the original and additional refined criteria and further narrowed down the subset to 47 articles. VD verified by going through 10% of the articles and the selection between the two reviewers was matching.

#### Inclusion criteria were

1. Peer-reviewed scientific literature.
2. Deals with implementation of state tobacco control policies from LMICs (World Bank classification of countries).
3. Describes and/or elaborates tobacco control policy implementation through at least one of the three strategies of the initial framework—awareness, enforcement and review systems; or explains microcontextual, mesocontextual, macrocontextual factors.
4. Discusses how and why of implementation: explains facilitators or barriers or contexts.

#### Exclusion criteria were

1. Material other than English and Indian regional languages.
2. Conference abstracts and dissertations.
3. Not focused on implementation of tobacco control policies but other aspects related to them for example, policy making, policy formulation.
4. Articles primarily quantifying implementation without explanators.
5. Articles with a primary disease-specific focus (cardiovascular, cancer, etc).
6. Programmes, projects, interventions that are not equivalent to state tobacco control policies.
7. Papers that are conceptual/theoretical assumptions or propositions of implementation of tobacco control policies, without empirical data.

### Appraisal of selected studies

The full-text quality assessment score was developed using the evaluative criterion prescribed by Giacomini and Cook,^18^ Mays and Pope,^19^ and was guided by the research question and IPT of this realist synthesis. A scoring system with a range of 0–5 was developed for assessing the rigour of the study and a range of 0–2 for assessing the relevance of the study to the research question. Each of the criteria was scored 1 if the criteria were met and 0 if that section/detail was missing in the full text. Articles with a quality score of 3 and above and relevance score of 1 and above were included for full-text analysis.

#### Quality

1. Clear objective/research question.
2. Sampling and overall research approach explained.
3. Data collection process explained.
4. Data analysis explained.
5. Coherence between objective—methods—findings.

#### Relevance

1. Explains—awareness/enforcement/review systems.
2. Explains microcontext, mesocontext or macrocontext.

### Data extraction, analysis and synthesis

PBH and VD imported the selected full-text articles were into NVivo (V.12) and coded them independently. The coding process was started with four codes from the IPT which were context, awareness, enforcement and review systems. Later, 12 codes arising from the data were added: advocacy; barriers; facilitators; gap in literature; gap in legislation/policy; institutionalisation; intersectoral coordination; mechanism—resources; mechanism—responses; role clarity; tobacco industry intereference and tobacco control strategies. On completing the independent coding process and intercoder discussion for all the articles we initiated data extraction using an article appraisal template (which PH learnt as part of masterclass on realist synthesis by Centre for Advancing Realist evaluation and synthesis, 2020). We extracted the article characteristics, reflections on the usefulness and relevance of the study findings and its strengths and weaknesses. Later, an Excel sheet was used to extract most relevant data that were contributing to the CMO configurations. Four major strategies emerged from the data which helped refine the IPT.

We organised a series of three workshops to synthesise the data iteratively . The first workshop included four early career researchers (with experience in realist methods), PBH and UB (with experience in tobacco control and knowledge about the data) and was facilitated by NSP (having relevant research experience in realist methods). The first workshop aimed to check the coherence of the CMOs developed for each of the four themes. During the second and third workshop, PBH and VD worked closely with the data to develop programme theory formulations and visualisations; UB contributed based on his experience of tobacco control and PNS the strength of realist methodology.

Data analysis techniques used in realist reviewing include reconciling, situating, adjudicating, juxtaposing and consolidating.^20^ In this realist synthesis, the data were used to situate implementation mechanisms in LMIC settings, juxtaposing new mechanisms to build on the IPT, and propose a refined programme theory to explain the implementation of tobacco control policies in LMIC settings.

## Results

### Article characteristics

The included articles (n=47) were published between 2007 and 2022, with more than half of the articles (n=29) published between 2016 and 2022. The articles described various policy measures with 17 of them addressing tobacco control policies in general, 11 of them addressing the implementation of the WHO FCTC, four examining smoke-free policies, three examining cessation services, and two each on tobacco advertising promotion and sponsorship, package warnings, and smokeless tobacco. The article characteristics are described in online supplemental file 3. While the geographical scope of this review was limited to LMICs, we noted that 27 of 47 articles had more than 50% of authors affiliated with high-income country institutions and only 12 articles had all authors affiliated with LMIC-based institutions.

10.1136/bmjgh-2022-008859.supp3 Supplementary data

We developed a total of 79 CMO configurations after detailed coding, reading and re-reading of the included articles. These CMOs were used in the workshops to frame 10 preliminary If-then propositions (programme theories) (see online supplemental file 4). The if-then propositions help explain narratively the theories through thick descriptions. Using the CMOs and the if-then propositions, four visualisations were developed to capture the working of the four strategies explained below.

10.1136/bmjgh-2022-008859.supp4 Supplementary data

### Awareness

**Figure 3:**
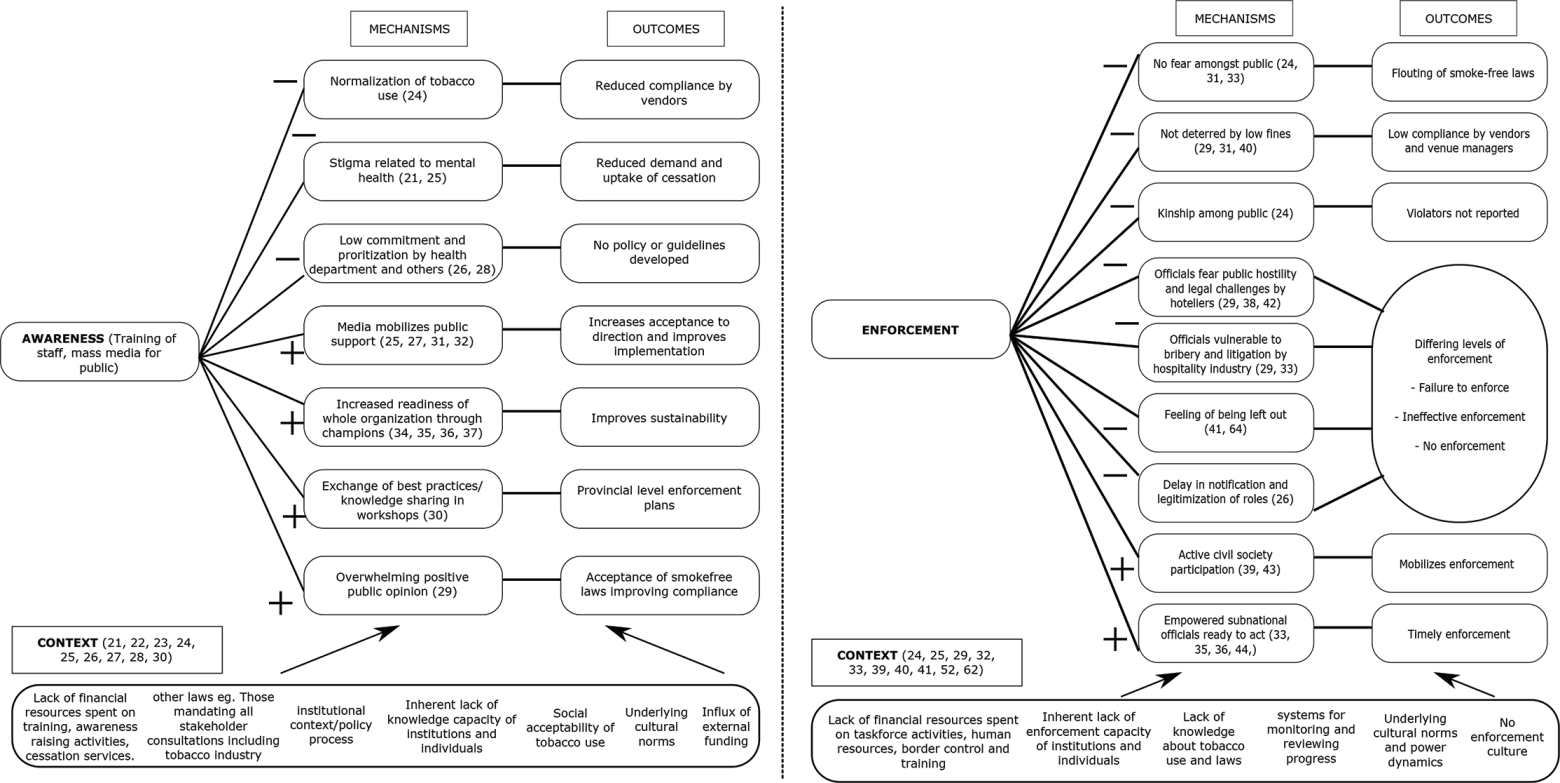
Context (C), mechanism (M), outcome (O) configurations related to awareness (left) and enforcement (right) IPTs. IPTs, initial programme theories.

The strategy of awareness consists of mass media attention, awareness-raising activities for the public, and training and capacity building for authorised personnel in charge of implementation. Twenty-four articles elaborated on the awareness strategy; eight of which discussed tobacco cessation. Figure 3 depicts the commonly found contextual factors in several articles such as individual and institutional capacity, knowledge and financial resources underlying sociocultural norms, other national laws and institutional context/policy processes.^21–30^ These factors interact with some of the mechanisms identified in the articles to produce negative outcomes (such as reduced compliance, reduced demand and reduced uptake of cessation services, and delay in implementation) and some positive outcomes (such as the development of enforcement plans, improved implementation and sustainability).

Seven mechanisms (facilitating and hindering) were found in the awareness strategy which are (1) normalisation, (2) stigmatisation, (3) commitment and prioritisation, (4) mobilisation, (5) organisational readiness through champions, (6) positive public opinion and (7) knowledge sharing. Normalisation of tobacco use was observed in the Gambia: parents there sent their children to run errands, including purchasing tobacco products. Vendors often do not ask for age proof, this affects the implementation of restriction of youth access to tobacco normalising the purchasing behaviour by children and lack of enforcement.^24^ In some LMICs, the stigma related to mental health impeded the uptake of cessation services as they were located in the same setting.^21^ The limited commitment of officials and the competing priorities of tobacco control with maternal and child health and other infectious diseases led to delay in developing implementation guidelines in the Pacific islands.^26 28^ Lack of awareness among the public impeded implementation by reduced cooperation and reduced demand for cessation interventions in India.^31^ The media can counter this by highlighting the issue of tobacco control and mobilising public support.^25 31 32^ Training of officials would empower them to counter the tobacco industry, engage civil society effectively, develop provincial level enforcement plans (in India). Training would help officials in accepting their role, improving the overall readiness of the whole organisation (as witnessed in Vietnam). Thus, capacitating them to raise further funding and improve sustainability while creating champions who can raise the issue of tobacco control in important fora.^32–37^ It was also noted that in some instances, knowledge about tobacco control may not translate to action if the structure does not involve knowledgeable actors into the implementation process.^38^

### Enforcement

The strategy of enforcement focused on the machinery of enforcement and how it could be executed. Eleven articles elaborated on the enforcement strategy. Figure 3 depicts some of the common contextual factors, such as a lack of financial resources for enforcement activities, lack of knowledge and capacity of staff and the system, underlying cultural norms and power dynamics, systems for monitoring and reviewing progress, tobacco industry interference, and the lack of an enforcement culture.^24 25 32 33 39–41^ These contextual factors interact with some of the mechanisms identified in the articles to produce outcomes such as flouting of the laws, failure to enforce the laws, and also positive outcomes such as timely enforcement.

Nine mechanisms were found in the enforcement strategy: (1) fear, (2) deterrence, (3) kinship, (4) hostility, (5) vulnerability, (6) legitimacy, (7) feeling of being left out, (8) civil society participation and (9) empowerment of officials. A lack of fear of enforcement was seen among the public and certain sectors (such as hospitality) in Uganda, where they did not have strict enforcement of smoke-free laws.^24 31 33^ In India, officials feared public opposition to enforcement; despite their knowledge on the matter, they hesitated to enforce the laws.^38^ While in the Eastern Mediterranean officials feared litigations and resistance from the hospitality sector if they would enforce the smoke-free law.^29 42^

Further, people in Egypt and Iran were not deterred by the low fines stipulated in the laws, thereby reducing the effect of enforcement.^29 31 40^ Another mechanism negatively impacting the enforcement of smoke-free laws was the sense of kinship in the Gambia which prevented people from complaining against offenders.^24^ Officials were unable to enforce the law unless their role was legitimised through notifications (government circulars or orders).^26^ Active civil society was seen to mobilise enforcement by government agencies in India and Mexico, whereas limited antitobacco advocacy by non-governmental organisations weakened enforcement in Pacific island countries.^37 39 43^ The susceptibility of officials in Uganda to bribery and resistance and the litigations from the hospitality sector in Eastern Mediterranean was all found to impact enforcement negatively.^33 42^ Contrarily, empowering subnational authorities in Iran, Nigeria, and community-based organisations in Bangladesh through diverse membership taskforces and mobile courts led to timely enforcement.^35 36 44^ In South Africa and Togo, ministries like law and justice, and the media felt left out of the policy-making process and were included only at the enforcement stage hence reducing their buy-in.^45^

### Intersectoral coordination

**Figure 4:**
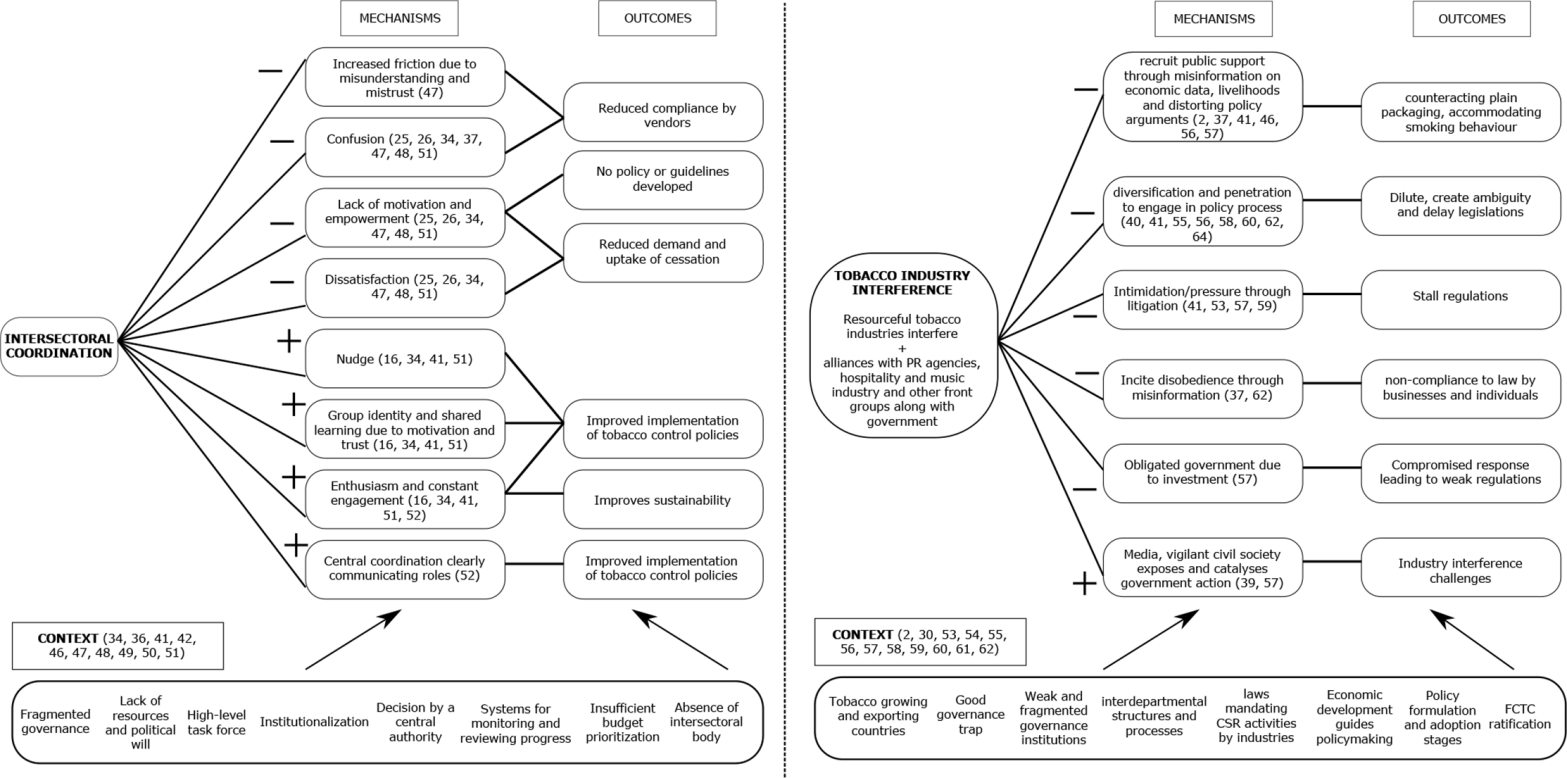
Context (C), mechanism (M), outcome (O) related to Intersectoral coordination (left) and tobacco industry interference (right) IPTs. IPTs, initial programme theories.

The strategy of intersectoral coordination examined coordination between health and other departments, as well as within the various levels or subsections of the health department itself. 19 articles discussed the intersectoral coordination strategy. Figure 4 depicts the common contextual factors seen in these articles, such as lack of resources and political will in emerging economies, fragmented governance, tobacco growing and exporting countries, top-down policy-making, change in political regimes and FCTC ratification.^34 41 46–51^ These interact with some of the mechanisms identified in the articles to produce outcomes such as delay and dilution of implementation efforts, interdepartment rivalry, poor staff retention and positive outcomes such as improved intervention uptake and sustainability.

Eight mechanisms were identified in the intersectoral coordination strategy, which were (1) friction, (2) confusion, (3) demotivation, (4) dissatisfaction, (5) nudge, (6) shared learning and group identity, (7) enthusiasm, and (8) central point of contact. Trust was observed in Vietnam, where a collective sense of responsibility was seen among the health department staff. In contrast, the issue of mistrust was observed in Kenya where siloed organisational cultures led to a misunderstanding or rivalry and friction as the actions were interpreted as either overstepping or underperforming.^47 51^ This misunderstanding in turn demotivated and disempowered staff as they were confused with the unclear goals and dissatisfied, which affected retention.^25 26 34 47 48 51^

Despite contextual challenges, in countries like Vietnam, India and Kenya, communication of a shared value by a legitimate central authority nudged government departments to align their priorities. It also helped to develop a group identity, increasing their enthusiasm and collective motivation. This enabled a positive working environment, thus improving sustainability of tobacco control policies in the long run.^16 34 41 51 52^

### Tobacco industry interference

The strategy of tobacco industry interference encompassed the various tactics used by resourceful tobacco industries to weaken national and local implementation efforts. Twenty-four articles discussed the tobacco industry interference strategy. Figure 4 depicts the common contextual factors such as policy formulation, interdepartmental structures or good governance trap, policy adoption stages in countries, fragile and unstable governments in LMIC settings and FCTC ratification.^2 30 53–61^ These interact with some of the mechanisms to produce outcomes such as dilution and delay in tobacco control policy formulation and weak implementation.

Six mechanisms were found in the tobacco industry interference strategy, which were those of (1) manipulation, (2) persuasion, (3) intimidation, (4) obligation, (5) inciting disobedience and (6) catalysation. Studies from Africa, India, Nepal and Latin America have shown how the tobacco industry manipulates government and public opinion by discrediting science, preempting actions and misinforming about revenue generation, hurting business, farmers livelihoods and persisting smuggling.^2 41 46 56 57 62^ The industry works to persuade public and policy makers directly as well as indirectly through liaising with advertisement agencies, music industries and the hospitality sector to dilute and delay legislations.^40 41 55 56 58 62 63^ Tobacco industry intimidates governments through large and continuous legal battles to align policies with their interests and stall the implementation of policies.^41 53 57 59^ In India, the use of the ‘right to information act’ by vigilant civil society organisations exposed the tobacco industry investments in the government, compromising the stance of the tobacco industry.^57^ Similarly in the Pacific Island countries and Nepal the role of media advocacy to expose instances of tobacco industry interference was crucial to catalysing government action.^39 62^

### Refinement of the IPT

**Figure 5:**
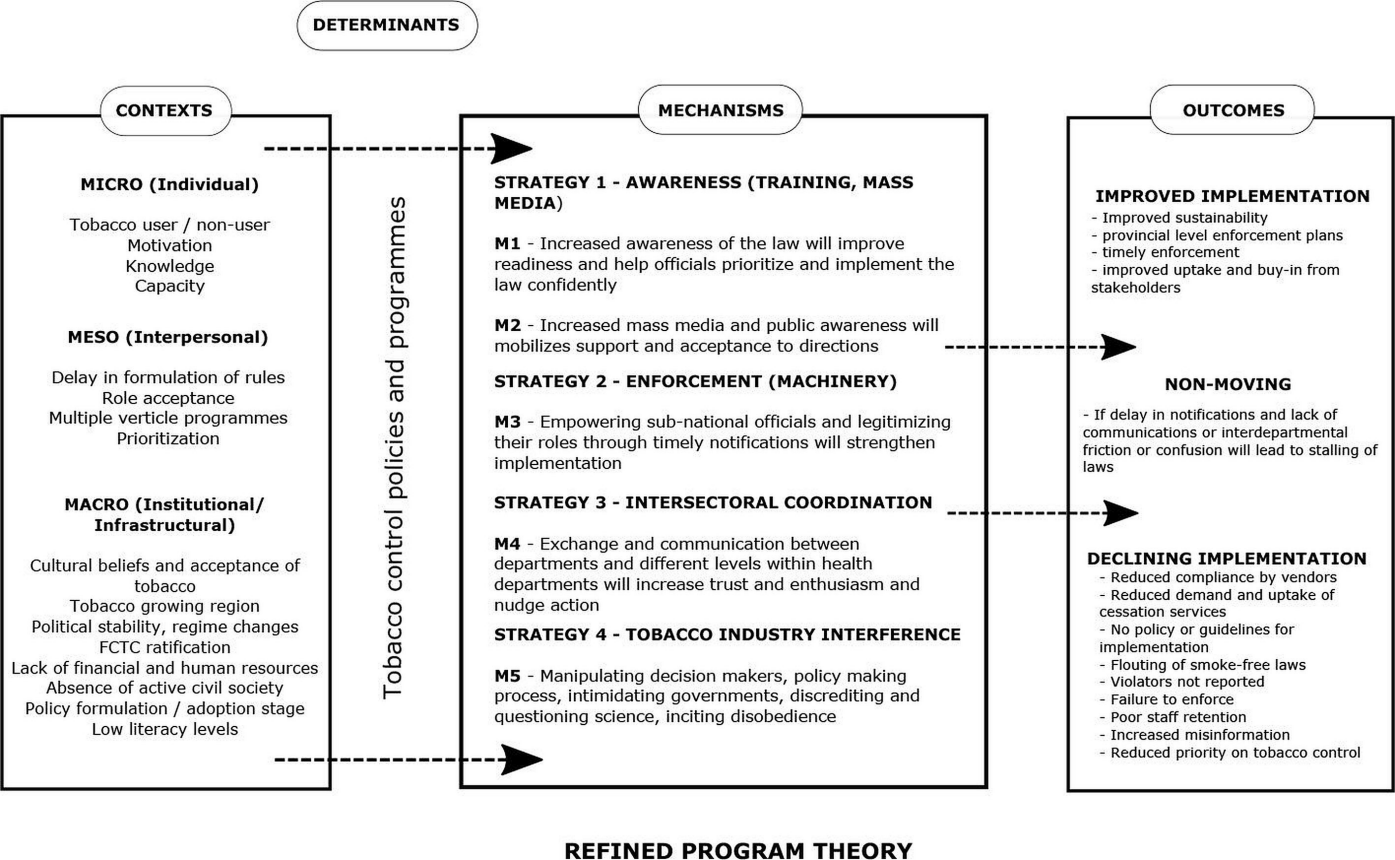
Schematic explaining the refined programme theory.

Based on the data analysis, the IPT was refined to shift some contextual factors into the strategies under which relevant mechanisms were found to be triggered (see figure 5). One of the proposed strategies (review systems) was not found to be well supported in the literature with only a few articles in India and neighbouring countries, such as Nepal and Bangladesh mentioning it. Two strategies (intersectoral coordination and tobacco industry interference) were part of the IPT as a context and were shifted to the mechanism in the refined programme theory. Since there was a lack of longitudinal studies in the selected articles, we cannot conclude causality for most mechanisms. All the mechanisms related to tobacco industry interference and some mechanisms related to intersectoral coordination (such as nudge) some mechanisms under awareness (such as stigma) have been well researched and documented. However, those related to enforcement require further research. Broadly, the strategy of awareness appeared to be working at a micro (individual) level, whereas the strategies of intersectoral coordination and enforcement were in the realm of mesolevel (interpersonal), and tobacco industry interference was working in the macrolevel (infrastructural/institutional).

## Discussion

**Figure 6:**
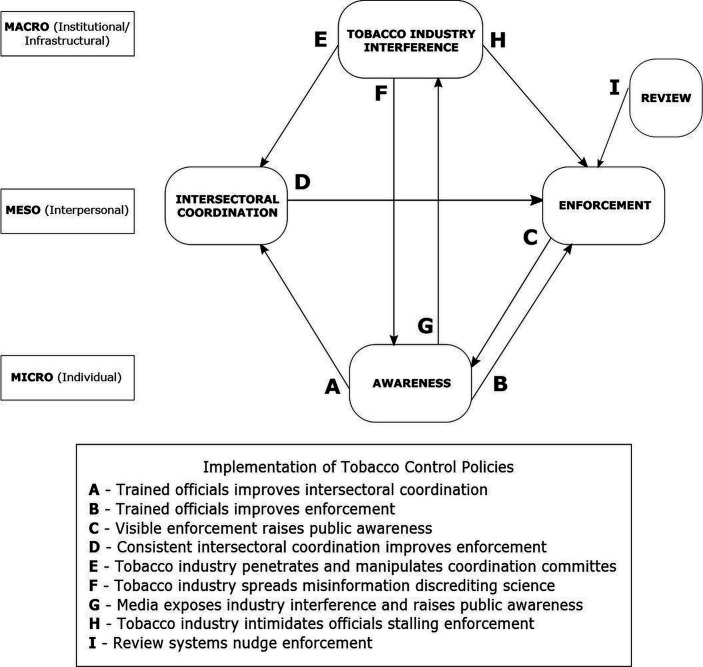
Implementation strategies for tobacco control policies in LMIC settings. LMIC, low-income and middle-income country.

The current study synthesises contextual factors present in various LMICs and explains mechanisms for how four major strategies of awareness, enforcement, intersectoral coordination and tobacco industry interference shape tobacco control policy implementation process and outcomes. The interplay of these strategies working across the three levels has been described in figure 6.

Limited financial and human resources, lack of political will was a common context to many of the LMICs,^21–26 28 33 39 41 42 52^ but there were also some unique barriers such as stigma of mental health impeding uptake of cessation services in India,^21^ lack of enforcement culture and kinship preventing reporting of offenders in the Gambia,^24^ and limited antitobacco advocacy by Non Governmental Organizations (NGOs) in the Pacific islands^39^ other laws mandating intersectoral consultations and corporate social responsibility activities by industries,^58–61^ which further hindered implementation of tobacco control policies. Whereas, other contextual factors facilitated implementation, such as ratification of FCTC which nudged countries to act,^34^ proactive civil society utilising the right to information act and media advocacy to mobilise government action,^37 43 62^and champions raising the issue of tobacco control in important forum despite resistance.^34 37 41 49^

Researchers, decision-makers as well as implementers working in LMIC settings on tobacco control can use these explanations to understand factors that are favourable in their context and those that require urgent emphasis to improve implementation of tobacco control policies. While knowledge about the law among the implementers is crucial, it may not always translate into action due to fear of opposition by the public or inadequate legitimisation and co-option into the implementation process. Enforcement often suffers from several practical challenges such as timely notifications and public support to encourage reporting of violators and making sure the enforcement is visible. Intersectoral coordination should begin during the policy formulation stage itself to improve buy-in of departments and continue throughout the implementation phase in a trustful and empowering manner. The tobacco industry tactics are documented, and evidence of preempting effective policies exists. Recognising and countering it would help vulnerable countries tackle this barrier to implementation.

While frameworks on what policies ought to be used for tobacco control such as WHO-FCTC at global level and national level laws exist, there ought to be guidance on how these policies could be better implemented, especially tailored to regional, national (and subnational) levels in LMICs. Here, our attempt at generating programme theories explaining implementation of tobacco control policies in LMICs would be of specific relevance. This realist synthesis with a geographical scope of LMICs will be followed by a realist evaluation of select tobacco control policies in India, which would help in testing and refining the programme theories.

Some of the strengths of this review include the diversity in the research team which consisted of individuals with experience from diverse socio-economic settings as well as varying professional backgrounds including epidemiology, medicine, public health, political science and communication. The workshop model followed during the data analysis phase helped to critically analyse the data. Some of the limitations were the inability to find a common database across LMIC settings to search for grey literature, hence we did not include grey literature in this synthesis except for review of policy documents to inspire the IPT development. This synthesis is as good as the vastness/depth of published implementation research literature in LMICs while being mindful of the kind of publication bias that exists.^64^ Since the review included peer-reviewed scientific literature published in English language the applicability of these findings would laregly be to ex English-colony LMICs that publish research in English. Also, as a variety of tobacco control policies exist, this study cannot provide specific deeper insights into each of them but rather provides an overview or a starting point for future realist studies. Realist philosophy and the conduct of a realist synthesis is time and resource intensive, it required capacity building of team members periodically and took approximately a year to complete. Researchers need to bear this in mind as they set out to undertake realist studies.

Future research into tobacco control policies such as smoke-free laws, pictorial warnings, taxation and testing of relevant mechanisms will help deepen the understanding of the implementation of tobacco control policies. Future research could attempt to deconstruct the contextual factors identified in this study to identify mechanisms that may be nested in them. Studies testing some of the IPTs to improve intersectoral coordination, awareness and enforcement in varying LMIC contexts could provide actionable guidance to officials involved in the implementation process. Such studies will provide the empirical basis that exists for the mechanisms related to tobacco industry interference.

## Data Availability

All data relevant to the study are included in the article or uploaded as online supplemental information. NA.
