# Supplementary material for: How do tobacco control policies work in low-income and middle-income countries? A realist synthesis
Source: BMJ Glob Health. 2022 Nov 8;7(11):e008859. doi: 10.1136/bmjgh-2022-008859 (PMC9644319; doi:10.1136/bmjgh-2022-008859)
Supplement: Supplementary data [file bmjgh-2022-008859supp001.pdf]

Article title: How do Tobacco Control Policies Work? Facilitators of and Barriers to Implementing Tobacco Control Policies in Low- And Middle-Income Countries: A Realist Synthesis

Journal name: BMJ Global Health

Authors' information: Pragati B Hebbar*, Vivek Dsouza, Upendra Bhojani, Prashanth Nuggehalli Srinivas, Onno C P van Schayck, Giridhara R Babu, Gera E Nagelhout.

1. Institute of Public Health, Bengaluru. pragati@iphindia.org

2. Institute of Public Health, Bengaluru

3. Institute of Public Health, Bengaluru.

4. Institute of Public Health, Bengaluru.

5. Department of Family Medicine, Maastricht University (CAPHRI)

6. Indian Institute of Public Health, Public Health Foundation of India (PHFI)

7. Department of Health Promotion, Maastricht University (CAPHRI)

Supplementary file 1: RAMESES checklist items

Table S1: List of items included when reporting a realist synthesis.

Reporting Item		Description of item	Reported on page(s)*
Title			
1		In the title, identify the document as a realist synthesis or review.	1
Abstract			
2		While acknowledging publication requirements and house style, abstracts should ideally contain brief details of: the study's background, review question or objectives; search strategy; methods of selection, appraisal, analysis and synthesis of sources; main results; and implications for practice.	1 – 2
Introduction			
3	Rationale for review	Explain why the review is needed and what it is likely to contribute to existing understanding of the topic area.	3
4	Objectives and focus of review	State the objective(s) of the review and/or the review question(s). Define and provide a rationale for the focus of the review.	4
Methods			

Hebbar PB, *et al. BMJ Global Health* 2023; 7:e008859. doi: 10.1136/bmjgh-2022-008859

5	Changes in the review process	Any changes made to the review process that was initially planned should be briefly described and justified.	None made
6	Rationale for using realist synthesis	Explain why realist synthesis was considered the most appropriate method to use.	3 – 4
7	Scoping the literature	Describe and justify the initial process of exploratory scoping of the literature.	4
8	Searching processes	While considering specific requirements of the journal or other publication outlet, state and provide a rationale for how the iterative searching was done. Provide details on all of the sources accessed for information in the review. Where searching in electronic databases has taken place, the details should include, for example, name of database, search terms, dates of coverage and date last searched. If individuals familiar with the relevant literature and/or topic area were contacted, indicate how they were identified and selected.	5 – 6
9	Selection and appraisal of documents	Explain how judgements were made about including and excluding data from documents and justify these.	6 – 7
10	Data extraction	Describe and explain which data or information were extracted from the included documents and justify this selection.	8 – 9
11	Analysis and synthesis processes	Describe the analysis and synthesis processes in detail. This section should include information on the constructs analyzed and describe the analytic process.	8 – 9
Results			
12	Document flow diagram	Provide details on the number of documents assessed for eligibility and included in the review, with reasons for exclusion at each stage, as well as an indication of their source of origin (e.g. from searching databases, reference lists and so on). You may consider using the example templates (which are likely to need modification to suit the data) that are provided.	8
13	Document characteristics	Provide information on the characteristics of the documents included in the review.	9 – 12
14	Main findings	Present the key findings with a specific focus on theory building and testing.	13 – 17
Discussion			
15	Summary of findings	Summarize the main findings, taking into account the reviews objective(s), research question(s), focus and intended audience(s).	18
16	Strengths, limitations and	Discuss both the strengths of the review and its limitations. These should include (but need not be	19

	future research directions	restricted to) (a) consideration of all the steps in the review process and (b) comment on the overall strength of evidence supporting the explanatory insights which emerged.<br>The limitations identified may point to areas where further work is needed	
17	Comparison with existing literature	Where applicable, compare and contrast the reviews findings with the existing literature (e.g., other reviews) on the same topic.	18 – 19
18	Conclusion and recommendations	List the main implications of the findings and place these in the context of other relevant literature. If appropriate, offer recommendations for policy and practice.	19
19	Funding	Provide details of funding source (if any) for the review, the role played by the funder (if any) and any conflicts of interests of the reviewers.	Yes

*Page no(s) subject to change based on acceptance and publication.

Hebbar PB, *et al. BMJ Global Health* 2023; 7:e008859. doi: 10.1136/bmjgh-2022-008859
