# Supplementary material for: How do tobacco control policies work in low-income and middle-income countries? A realist synthesis
Source: BMJ Glob Health. 2022 Nov 8;7(11):e008859. doi: 10.1136/bmjgh-2022-008859 (PMC9644319; doi:10.1136/bmjgh-2022-008859)
Supplement: Supplementary data [file bmjgh-2022-008859supp002.pdf]

MY EBSCOhost

Monday, May 09, 2022 9:21:06 AM

#	Query	Limiters/Expanders	Last Run Via	Results
S1	(tobacco) AND (polic* OR law OR act OR statut* OR regulat* OR ban* OR restrict*) AND (implement* OR enforce* OR compliance* OR adher*) AND (Afghanistan OR Albania OR Algeria OR American Samoa OR Angola OR Argentina OR Armenia OR Azerbaijan OR Bangladesh OR Belarus OR Belize OR Benin OR Bhutan OR Bolivia OR “Bosnia and Herzegovina” OR Botswana OR Brazil OR Bulgaria OR Burkina Faso OR Burundi OR “Cabo Verde” OR Cambodia OR Cameroon OR “Central African Republic” OR Chad OR China OR Colombia OR Comoros OR “Congo” OR “Costa Rica” OR “Cote d’Ivoire” OR Cuba OR Djibouti OR Dominica OR “Dominican Republic” OR Ecuador OR Egypt OR “El Salvador” OR “Guinea” OR Eritrea OR Eswatini OR Ethiopia OR Fiji OR Gabon OR Gambia OR Georgia OR Ghana OR Grenada OR Guatemala OR Guinea OR Guinea-Bissau OR Guyana OR Haiti OR Honduras OR	Expanders - Apply equivalent subjects<br>Search modes - Boolean/Phrase	Interface - EBSCOhost Research Databases<br>Search Screen - Advanced Search<br>Database - CINAHL;EconLit;ERIC;Library, Information Science & Technology Abstracts;APA PsycArticles;APA PsycInfo;SocINDEX;MEDLINE;APA PsycBooks;eBook Collection (EBSCOhost);OpenDissertations;eBook Academic Collection (EBSCOhost);Health and Psychosocial Instruments	3,393

India OR Indonesia OR Iran OR Iraq OR Jamaica OR Jordan OR Kazakhstan OR Kenya OR Kiribati OR Korea OR Kosovo OR “Kyrgyz Republic” OR “Lao PDR” OR Lebanon OR Lesotho OR Liberia OR Libya OR Madagascar OR Malawi OR Malaysia OR Maldives OR Mali OR “Marshall Islands” OR Mauritania OR Mauritius OR Mexico OR “Micronesia” OR Moldova OR Mongolia OR Montenegro OR Morocco OR Mozambique OR Myanmar OR Namibia OR Nepal OR Nicaragua OR Niger OR Nigeria OR “North Macedonia” OR Pakistan OR Panama OR “Papua New Guinea” OR Paraguay OR Peru OR Philippines OR Romania OR “Russian Federation” OR Rwanda OR “Samoa Sao Tome and Principe” OR Senegal OR Serbia OR “Sierra Leone” OR “Solomon Islands” OR Somalia OR “South Africa” OR “South Sudan” OR “Sri Lanka” OR “St. Lucia” OR “St. Vincent and the Grenadines” OR Sudan OR Suriname OR “Syrian Arab Republic” OR Tajikistan OR Tanzania OR Thailand OR Timor-Leste OR Togo OR Tonga OR Tunisia OR Turkey OR Turkmenistan OR Tuvalu OR Uganda OR Ukraine OR

Supplemental material
BMJ Publishing Group Limited (BMJ) disclaims all liability and responsibility arising from any reliance placed on this supplemental material which has been supplied by the author(s)

Uzbekistan OR Vanuatu OR Vietnam OR “West Bank and Gaza” OR Yemen OR Zambia OR Zimbabwe OR "low and middle income countries" OR "low income countries" OR "middle income countries")

Hebbar PB, *et al. BMJ Global Health* 2023; 7:e008859. doi: 10.1136/bmjgh-2022-008859
