# Supplementary material for: How do tobacco control policies work in low-income and middle-income countries? A realist synthesis
Source: BMJ Glob Health. 2022 Nov 8;7(11):e008859. doi: 10.1136/bmjgh-2022-008859 (PMC9644319; doi:10.1136/bmjgh-2022-008859)
Supplement: Supplementary data [file bmjgh-2022-008859supp003.pdf]

Article title: How do Tobacco Control Policies Work? Facilitators of and Barriers to Implementing Tobacco Control Policies in Low- And Middle-Income Countries: A Realist Synthesis

Journal name: BMJ Global Health

Authors' information: Pragati B Hebbar*, Vivek Dsouza, Upendra Bhojani, Prashanth Nuggehalli Srinivas, Onno C P van Schayck, Giridhara R Babu, Gera E Nagelhout.

1. Institute of Public Health, Bengaluru and Department of Health Promotion, Maastricht University (CAPHRI) pragati@iphindia.org

2. Institute of Public Health, Bengaluru.

3. Institute of Public Health, Bengaluru.

4. Institute of Public Health, Bengaluru.

5. Department of Family Medicine, Maastricht University (CAPHRI)

6. Indian Institute of Public Health, Public Health Foundation of India (PHFI)

7. Department of Health Promotion, Maastricht University (CAPHRI)

Supplementary file 3: Article characteristics of studies reviewed

First author (last names) and year of publication	Type of publication	Location of study	Contextual elements explained in the article	Strategy discussed in the article
Abu-Rmeileh et al 2022	Review	Eastern Mediterranean region	Macro, meso	Awareness
Banks C et al 2017	Review	Egypt and Iran	Macro, meso	Tobacco industry intereference
Bhatta ND et al 2020	Original research	Nepal	Macro, meso	Tobacco industry interference
Borges et al 2020	Review	Brazil	Macro	Tobacco industry interference

Bump et al 2013	Review	LMIC	Macro	Tobacco industry interference, intersectoral coordination
Byron et al 2019	Review	LMIC	Macro	Tobacco industry interference, intersectoral coordination
Carroll et al 2016	Review	Global	Macro, meso	Awareness, tobacco industry interference
Chugh A et al 2020	Review	India	Macro, meso	Tobacco industry interference
Craig et al 2019	Original research	12 countries	Macro, meso	awareness, tobacco industry interference
Crosbie E et al 2011	Original research	Mexico	Meso, micro	Awareness, enforcement
Egbe et al 2019	Review	Nigeria	Meso	Tobacco industry interference, awareness, enforcement
Elsey et al 2016	Original research	Nepal	Macro	awareness, review system
Ghose et al 2019	Original research	India	Macro, meso	awareness
Hebbar et al 2017	Original research	India	Macro, meso	Intersectoral coordination, review system
Hiilamo et al 2015	Short report	LMIC	Macro	Tobacco industry interference
Hirpa S et al 2022	Original research	Ethiopia	Meso, micro	Tobacco industry intereference, awareness,

				intersectoral coordination
Huque et al 2017	Review	Bangladesh	Macro, meso	Enforcement, awareness
Jackson-Morris et al 2015	Original research	Bangladesh	Macro, meso	awareness, intersectoral coordination, review system
Jagdish et al 2012	Short communication	India	Macro, meso	awareness
Jallow et al 2019	Original research	The Gambia	Macro	awareness
Kashiwabara et al 2011	Review	India	Macro, meso	awareness, review system, tobacco industry interference
Khan et al 2014	Brief report	South Asia	Macro	Enforcement, awareness
Kumar P et al 2022	Original research	India	Macro, meso	Tobacco industry interference, awareness, intersectoral coordination
Lencucha et al 2016	Original research	Zambia	Macro, meso	intersectoral coordination
Lencucha et al 2016	Original research	Kenya	Micro, meso	intersectoral coordination
Martin et al 2013	Original research	Pacific islands	Micro, meso	Enforcement, awareness, intersectoral coordination
McCool et al 2013	Original research	Pacific islands	Macro, meso	awareness, tobacco industry interference

Menon 2018	Original research	India	Micro, meso	Intersectoral coordination
Mohamed et al 2018	Original research	Kenya	Micro, meso, macro	Tobacco industry interference, intersectoral coordination
Mohan et al 2013	Short communication	India	Micro, meso	Intersectoral coordination, enforcement
Nagler et al 2013	Review	Global	Macro	Tobacco industry interference, enforcement, review system
Oladele et al 2013	Original research	Nigeria	Macro	awareness, tobacco industry interference
Oswal et al 2010	Review	India	Meso	Tobacco industry interference
Owusu-Dabo et al 2010	Original research	Ghana	Macro, meso	Tobacco industry interference, enforcement
Patel et al 2009	Original research	Africa	Macro	Tobacco industry interference, enforcement
Persai et al 2016	Original research	India	Micro, meso, macro	awareness, intersectoral coordination, review system
Robertson et al 2018	Original research	Uganda	Macro	Tobacco industry interference, enforcement
Sankaran et al 2015	Original research	India	Macro	Tobacco industry interference

Sanni et al 2018	Original research	Africa	Macro	Tobacco industry interference, intersectoral coordination
Sarrafzadegan et al 2016	Original research	Iran	Meso	Awareness, intersectoral coordination
Sebrie et al 2007	Original research	Latin America	Macro	Tobacco industry interference
Sharma et al 2017	Brief report	India	Micro, meso, macro	awareness, intersectoral coordination
Shelley et al 2019	Original research	Global	Macro	awareness
Singh A et al 2020	Review	Ghana	Meso, micro	Awareness, enforcement
Stillman et al 2014	Original research	Vietnam	Macro	awareness, intersectoral coordination, review system
VanDevanter et al 2017	Original research	Vietnam	Macro	intersectoral coordination
Wisdom et al 2018	Original research	Africa	Macro	intersectoral coordination
