# Supplementary material for: How do tobacco control policies work in low-income and middle-income countries? A realist synthesis
Source: BMJ Glob Health. 2022 Nov 8;7(11):e008859. doi: 10.1136/bmjgh-2022-008859 (PMC9644319; doi:10.1136/bmjgh-2022-008859)
Supplement: Supplementary data [file bmjgh-2022-008859supp004.pdf]

Article title: How do Tobacco Control Policies Work? Facilitators of and Barriers to Implementing Tobacco Control Policies in Low- And Middle-Income Countries: A Realist Synthesis

Journal name: BMJ Global Health

Authors' information: Pragati B Hebbar*, Vivek Dsouza, Upendra Bhojani, Prashanth Nuggehalli Srinivas, Onno C P van Schayck, Giridhara R Babu, Gera E Nagelhout.

1. Institute of Public Health, Bengaluru and Department of Health Promotion, Maastricht University (CAPHRI) pragati@iphindia.org

2. Institute of Public Health, Bengaluru.

3. Institute of Public Health, Bengaluru.

4. Institute of Public Health, Bengaluru.

5. Department of Family Medicine, Maastricht University (CAPHRI)

6. Indian Institute of Public Health, Public Health Foundation of India (PHFI)

7. Department of Health Promotion, Maastricht University (CAPHRI)

Supplementary file 4: Initial Program Theory (IPT) formulations using IF…THEN statements.

AWARENESS

a) Cessation (References: 21 – 25, 28, 51)

- **IF** there is provision of culturally appropriate messaging around tobacco harms through sustained mass media campaigns(25) along with presence of conducive and dedicated tobacco cessation counselling spaces(22)
THEN the utilization of cessation services will be improved
BECAUSE such campaigns help counter deep rooted cultural norms normalizing tobacco, prompting tobacco users to seek cessation help.(21, 22) The cessation facilities become more accessible and acceptable when they are separated from 'mental health' facilities reducing stigma for the service users.(21)

- **IF** providers of tobacco cessation services many of whom may be tobacco users themselves receive sustained and culturally appropriate training in a context where cost-effective cessation interventions receive increased government funding(23) and are regularly monitored(21, 22)
THEN the implementation coverage of cessation services will be improved
BECAUSE the enhanced user demand for cessation services, and the subsequent political priority for cessation services, mobilizes drafting of policies and guidelines around tobacco cessation.(25, 28) Training boosts the confidence and motivation of cessation service providers, by enhancing internalization of their roles/duties and healthcare

Hebbar PB, *et al. BMJ Global Health* 2023; 7:e008859. doi: 10.1136/bmjgh-2022-008859

providers spend more time with users delivering tailored cessation services/strategies.(22 – 24)

b) Awareness about tobacco control use and the law (References: 21, 23 – 27, 29, 31, 32, 33, 42, 44)

- **IF** the knowledge and awareness about the ill effects of tobacco use, as well as about the tobacco control law, is very low among the public officials mandated to enforce tobacco control laws(21, 23, 31)
  THEN the enforcement of the law would be suboptimal
  BECAUSE enforcement officers are not confident, lack role clarity, are unable to engage civil society resources, and are unable to counter legal challenges and tobacco industry interference.(25, 27, 29, 33, 42) Despite enforcement officials perceiving positive role of mass media in creating awareness(27), there remains lack of government and external funding in certain settings, for mass media campaigns due to lack of commitment and prioritization from health and other stakeholders.(25, 26, 44)

- **IF** the knowledge and awareness about the ill effects of tobacco as well as about the tobacco control law is very low among the general public(21, 23, 31)
  THEN youth tobacco use continues while cooperation and compliance from tobacco vendors(21), hoteliers(29, 42) and citizens in general for tobacco control law is hindered(31, 32)
  BECAUSE students and youth in general find anti-tobacco messages by government repulsive and scary(23) while continuing with positive perception of cigarettes influenced by models and film stars. Tobacco use is further normalized for youth as tobacco vendors do not ask for age proof and parents often send their kids to purchase tobacco products for them(24) wherein tobacco users tend to locate guilt for their addiction onto themselves or diabolism but not the tobacco companies.

b) Capacity building of stakeholders (References: 25, 27, 32 – 34, 48)

- **IF** regular capacity building and advocacy workshops are organized by the government using internal or external resources for health staff and enforcement officials(32, 34) with tailored training materials(48) in a context where inherent tobacco control capacity of individuals as well as institutions is low(25, 27, 34)
  THEN it leads to increased demand for further training at provincial levels(32) and enhances readiness of the entire organization to tobacco control, thereby improving sustainability of tobacco control initiatives(34 – 37)
  BECAUSE it leads to formulation of local implementation pathways, provincial enforcement action plans(32) and staff and local champions whose capacity is built are able to raise further financial resources and mainstream tobacco control at provincial level as well as other important fora despite resistance(27, 34) as they are empowered to

counter tobacco industry and involve the civil society and engage with relevant stakeholders.(33)

ENFORCEMENT

a) Public response to enforcement (References: 24, 25, 29, 33, 40)

- **IF** people are unaware of specifics of tobacco control laws including penalties, and do not (visibly) perceive strict enforcement of these laws, in a context where prevails a general lack of an enforcement culture(24, 33)
  THEN the compliance to the law is hampered and the law is commonly flouted by the public as well as street (tobacco) vendors(24, 29, 33, 40)
  BECAUSE the people do not fear or anticipate enforcement and are not deterred by the low fines stipulated in the law.(29, 40) Violations of law do not get reported either because of the underlying belief of kinship within the communities, preventing individuals from reporting offenders(24, 33) or because of hesitation to offend customer in hotels/trade settings.(25)

b) Officials' response to enforcement (References: 24 – 26, 32, 33, 38 – 41, 43, 44)

- **IF** financial and human resources are not invested into enforcement activities including capacity building(41), regular visits and inspections(39, 40), border control (24), communication of the law and penalties to the public
  THEN enforcement of laws remains poor leading to impeding compliance to the laws(33)
  BECAUSE enforcement officials lack knowledge and capacity because of their limited participation in policy formulation and perceiving tasks to be out of their jurisdiction(25, 32, 33, 39 – 41), sometimes hesitate to act fearing hostility from public despite the knowledge(38), are unable to conduct regular and adequate compliance checks(40, 41), are susceptible to bribery, litigations from hospitality sector(29, 33, 42), delay in notifying regulations or empowering sub-national authorities and community based organization to address violation in a timely manner(26, 44), lack of advocacy by civil society to mobilize government action.(32, 43)

INTERSECTORAL COORDINATION

a) Facilitator (References: 16, 34, 41, 51)

- **IF** multi-stakeholder consultation for tobacco control is institutionalized(41) wherein a high-level steering committee (task force) is established, a national public authority delivers decisions in line with health priorities(34), and a reporting system is in place(16) in contexts where decentralized institutions lack transparency and are overburdened(34, 41, 51)
  THEN it facilitates the formulation and implementation of TCPs due to sustained policy processes and improved uptake of interventions

BECAUSE communicating the value of a shared approach to tobacco control by a legitimate authority nudges senior government departments to align their priorities by helping them understand their roles and responsibilities(16, 34, 41); working together allows departments to communicate, learn from each other, and develop socio-political and cultural sensibilities which establishes a group identity(51); regular constructive feedback encourages ownership, fosters collective responsibility, and builds a sense of trust between departments which leads to a positive working environment(51) as stakeholders work, plan, and allocate resources together(41) in a team which in turn facilitates buy-in for implementation.(16) This increases the enthusiasm and collective motivation of junior staff which leads to sustainability in the long run.

b) Barrier (References: 16, 25, 26, 34, 41, 46 – 48, 51)

- **IF** a national intersectoral body is absent(46) and tobacco control financing for health and non-health staff recruitment and training as well as health interventions is inadequate(47) in countries wherein top-down governance institutions with differing mandates are under-resourced(16, 46, 51), have poor information exchange across departments, and are forced to rely on external funding(34, 41, 47)
  THEN formulation and implementation of comprehensive tobacco control policies gets delayed
  BECAUSE siloed organizational cultures lead to misunderstanding about intentions and mistrust between higher-up government departments leading to cross-department rivalry and friction as they perceive others' actions as overstepping or underperforming and do not perceive the value of a shared contribution to tobacco control.(47) As a result lower-level staff lack commitment and are demotivated and disempowered(25, 26, 47) to prioritize tobacco control as they are unable to handle multiple programs due to increasing workloads(48); inadequate/poor government wages increases staff dissatisfaction(34); and lack of clarity in providing NCD/tobacco control services due to unclear goals leads to confusion(47) between agencies as health staff are only familiar in treating infectious diseases(51) which in turn contributes to poor/low permanent staff retention.(34)

TOBACCO INDUSTRY INTERFERENCE

a) Barrier (References: 2, 40, 41, 45, 46, 49, 53 – 57, 63)

- **IF** there are alliances between resourceful tobacco industries(40, 41) and trade/commerce related public agencies as well as front groups (like Ad agencies, pro-tobacco NGOs, hospitality, and music industries)(40, 41, 55 – 57, 63) in countries wherein economic development is the main priority(46, 49) while financial/legal resources and advocacy for tobacco control are limited(49, 54) and governments fall into good governance traps through interdepartmental structures and processes(30, 58, 59, 61)
  THEN tobacco control policy is likely to be diluted and delayed resulting in weak implementation

BECAUSE tobacco industries manipulate government and public opinion through campaigns that misrepresent smoking as a positive activity and discredit science which creates an atmosphere of misinformation.(2, 41, 46, 56); Inciting disobedience from the individuals and businesses;They stall regulations by pushing for getting different varieties of tobacco products under legislation(57), intimidate governments through arguments and legal battles(41, 53) while their strong relationships convince legislators to align and invest(50) with industries by arguing that tobacco, as a cash crop, helps in revenue generation(2, 46) and tobacco control hurts farmers, businesses and leads to smuggling(2, 45); penetrating policy making processes (58, 60, 62); Health ministries' lack political power and will(2) and are unable to garner support from the whole of government due to lack of resources, weak leadership, diverse mandates related to tobacco and management across sectors(46) while ministries like law, justice, and media feel left out in policymaking(45) which in turn leads to a lack of prioritization of tobacco control by governments at the policy level.(55)
